# An automated high-throughput enterovirus D68 microneutralization assay platform

**DOI:** 10.1016/j.jviromet.2022.114590

**Published:** 2022-07-22

**Authors:** Eric E. Rhoden, Bernardo A. Mainou, Jennifer L. Konopka-Anstadt, M. Steven Oberste

**Affiliations:** Division of Viral Diseases, Centers for Disease Control and Prevention, Atlanta, GA, USA

**Keywords:** Enterovirus D68 (EV-D68), Acute flaccid myelitis (AFM), Virus neutralization assay, Viral cytopathic effect (CPE), Cell culture 50 % infectious dose, CCID_50_

## Abstract

Virus neutralization assays, widely used to detect and quantify antibodies induced by virus infection, are considered the gold standard for enterovirus serology testing. Conventional microneutralization assays have been used to assess enterovirus D68 (EV-D68) seroprevalence. While manual or automated 96-well assays are valuable, higher-density assays that increase throughput provide the opportunity to more efficiently screen large, population-based serology collections, as well as to test sample sets against multiple virus strains on the same plate or within the same run. Here, automation was implemented for bulk reagent dispensing, serial dilutions, and luminescence measurement to develop a 384-well enterovirus microneutralization assay that increases overall testing throughput, maintains the reproducibility of the standard 96-well assay, and reduces sample volume usage. EV-D68 strains Fermon, 14–18953, and 18–23087 were used to evaluate the automated 384-well microneutralization assay and compare to the conventional 96-well assay. Sensitivity and specificity were evaluated using pooled human sera and positive and negative control antisera. The Lower Limit of quantitation (LLOQ) was the same as for the 96-well assay and coefficients of variations (CV) of 7.35 %, 5.97 %, and 2.85 % for the three EV-D68 strains respectively, were well below the typical goal of ≤ 20 % CV for accuracy. Z-factor analysis yielded results of 0.694, 0.638, and 0.852, for the three EV-D68 strains respectively, indicating a high level of precision, reliability, and robustness. Intra-assay (7.25 %) and inter-assay (7.12 %) variability were well below 20 % CV. Moreover, the 96-well and 384-well versions of the assay were highly concordant, with a 0.955 correlation coefficient in titers obtained for 50 sera tested. Validation of this automated 384-well microneutralization will support its use in large serology screens assessing the presence of EV-D68 neutralizing antibodies in human populations.

## 1. Introduction

Enteroviruses are among the most common pathogens infecting infants and children (Coyne et al., 2021). Enterovirus D68 (EV-D68), primarily a respiratory pathogen, was first detected in children with pneumonia and bronchiolitis in 1962 (Schieble et al., 1967). Most EV-D68 infections are asymptomatic or mild, with reports of severe disease remaining relatively rare until the fall of 2014, when the U.S. experienced a nationwide outbreak of severe respiratory illness associated with EV-D68 (Midgley et al., 2015). Concurrently, cases of acute flaccid myelitis (AFM) cases were also reported, particularly afflicting young children (Sejvar et al., 2016).

AFM is a serious neurologic condition characterized by rapid-onset limb weakness or paralysis in otherwise healthy children (Sejvar et al., 2016). Since 2014, nationwide U.S. surveillance has demonstrated sharp increases in AFM cases every two years, with the exception of 2020, with some cases continuing to occur coincident to outbreaks of enterovirus-associated respiratory disease (Kidd et al., 2020; Park et al., 2021). Despite these trends, diagnostic testing has rarely detected a pathogen in the cerebrospinal fluid (CSF) of AFM patients, making it challenging to conclusively pinpoint the etiology of AFM (Kidd et al., 2020). However, data accumulated over the past six years support enterovirus infection, including EV-D68, as a contributing factor in AFM (Messacar et al., 2018). New avenues of research have sought to identify indirect evidence of infection occurring prior to the onset of AFM. For example, AFM patients were more likely than non-AFM patients to have enterovirus-specific antibodies present in CSF, supporting a role for recent enterovirus infection as an underlying factor for AFM (Mishra et al., 2019; Schubert et al., 2019).

Neutralizing antibodies (nAb) are generally regarded as the primary correlate of protection from enterovirus disease, particularly exemplified by poliomyelitis (McKay et al., 1963; Mermel et al., 1993). While specific factors mediating protection from EV-D68 respiratory disease have not been directly demonstrated in humans, anti-enterovirus antibodies can protect against paralysis and death in murine models of EV-D68 neural infection, supporting the induction of humoral immunity as a key protective factor against EV-D68-associated disease (Hixon et al., 2017; Morrey et al., 2018; Zhang et al., 2018). Despite the important role of antibodies in the outcome of enterovirus infection, levels of EV-D68-specific neutralizing antibodies in the general U.S. population have not been well characterized. A single serosurvey performed in the U.S. demonstrated that antibodies capable of neutralizing EV-D68 were widely present in both adults and children prior to the 2014 outbreak of respiratory disease. However, this study was small in scope and not population-based. Larger, population-based studies have not been feasible with the existing 96-well assay.

Here, we describe an automated high-throughput microneutralization assay that provides a platform with high reproducibility and quantitative output for the screening of EV-D68-specific nAb in human serum specimens against various EV-D68 strains. The assay is a modification of the dried blood spot (DBS) poliovirus microneutralization assay that uses a 384-well plate format and automated liquid handling (Weldon et al., 2016). The 384-well platform also allows for conservation of specimens, particularly valuable in testing specimen collections with limited volume available or precious specimen types. A robust, automated, highly reproducible, and quantitative assay that also reduces the amount of sample required for testing will be critical to complete wide-scale EV-D68 serosurveys. This platform also can easily be modified for future high-throughput screening, including identification of small molecules with antiviral effects on EV-D68 infection.

## 2. Materials and methods

### 2.1. Cells, viruses, and control antisera

EV-D68 reference strain Fermon (GenBank NC038308.1) and Coxsackievirus B5 (Faulkner) were originally obtained in the 1970 s as an NIH Reference Reagent (Bethesda, MD). EV-D68 strain 14–18953 (subclade D1, GenBank KX957754.1) was isolated in 2014 from a nasopharyngeal swab from a patient (USA/KY) with respiratory illness. EV-D68 strain 18–23087 (subclade B3, GenBank MK491180.1), isolated in 2018 from a nasopharyngeal swab of a human subject (USA/CO) and EV-D70 reference strain J670/71 (GenBank MT087378.1), were amplified from in-house stocks. RD cells (human rhabdomyosarcoma; ATCC CCL-136) were maintained in minimal essential medium (MEM; Gibco, Grand Island, NY) with Earle’s salts and 10 % fetal bovine serum (FBS; HyClone, Logan, UT). Poliovirus type 1 (Sabin vaccine strain) was obtained from the National Institute of Biological Standards and Control (Potters Bar, UK). Enterovirus A-71 strain SH-17 was provided by Dr. Wenbo Xu, China Center for Disease Control and Prevention, Beijing, China. Horse EV-D68-specific antiserum (T2–3420) and pooled human in-house reference sera (IHRS) served as positive serology controls. Irrelevant rabbit antisera raised against EV-D70 strain J670/71 served as a negative EV-D68 serology control.

## 3. EV-D68 microneutralization assay

### 3.1. Serial dilutions of serum samples

**Fig. 1.**
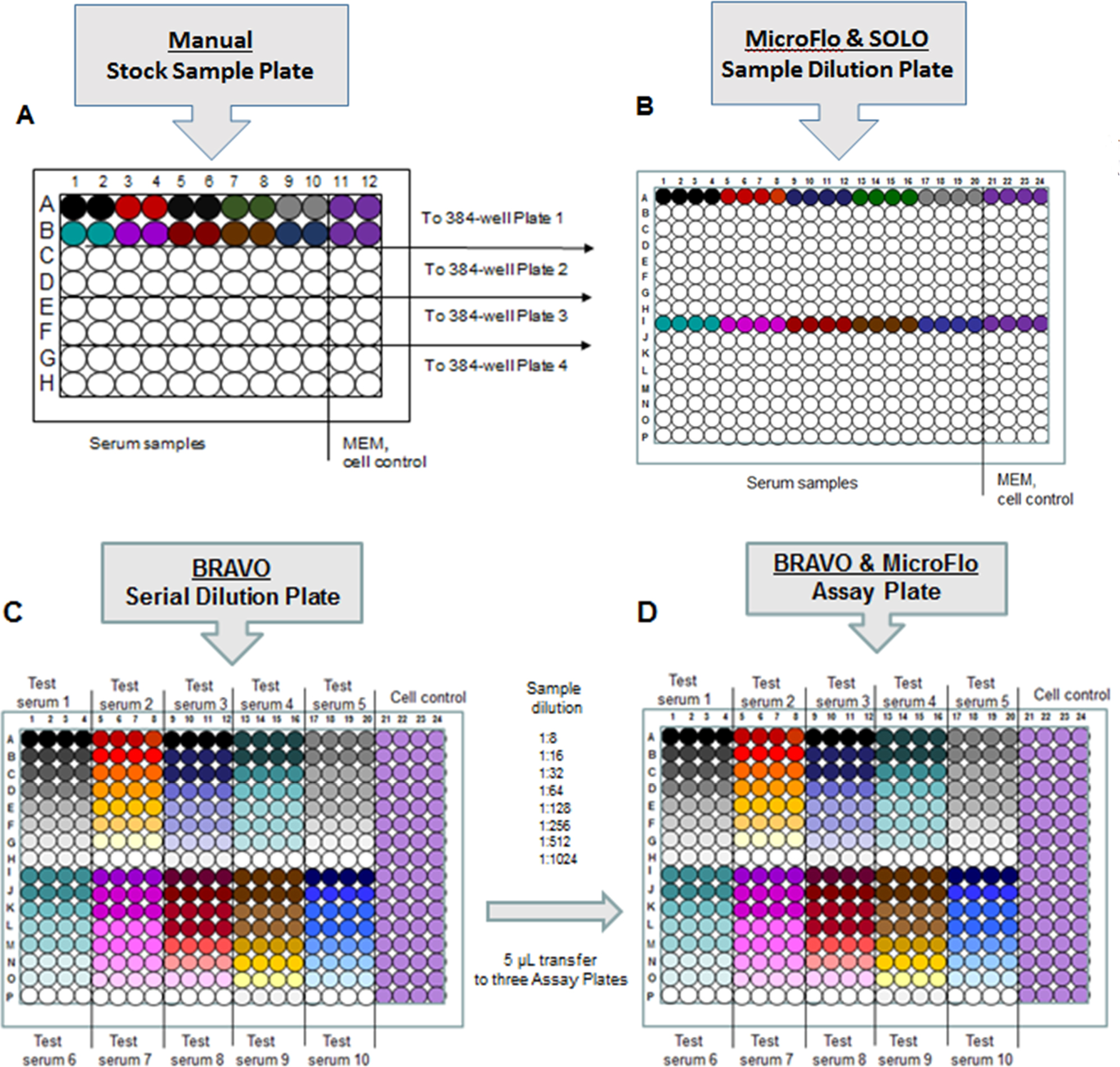
Workflow for EV-D68 microneutralization assay. A. Preparation of Stock Sample Plate: Manual serum sample transfer by pipet from sample vials into four 96-well V-bottom microplates. B. Preparation of Sample Dilution Plate: MicroFlo^™^ Select dispenser is used to add 30 μL of media (MEM + 2 % FBS) to all wells of a white, 384-well, flat-bottom plate (the Sample Dilution Plate). SOLO multi-channel robotic workstation is then used to transfer 30 μL of the diluted stock serum in quadruplicate from the Stock Dilution Plate into rows A and I of the 384-well Sample Dilution Plate (only “Plate 1” shown). C. Creation of a Serial Dilution Plate: Bravo Liquid Handling Platform executes 0.5 log_2_ serial dilutions (30 μL total volume) to transform a Sample Dilution Plate into a Serial Dilution Plate. D. Completing an Assay Plate: Bravo Liquid Handling Platform transfers 5 μL of each serum dilution from the Serial Dilution Plate into three separate 384-well assay plates, with each Assay Plate separately assessing three distinct EV-D68 strains.

The 96-well microneutralization assay was performed as described (Weldon et al., 2016). For the 384-well microneutralization assay, serum specimens were manually transferred (25 μL) from 2 mL cryovials in duplicate to columns 1 through 10 of a 96-well, V-bottom polystyrene plate (Stock Sample Plate, Fig. 1A). For control wells, 25 μL of MEM + 2 % FBS was added in duplicate to columns 11 and 12 (Fig. 1A). A MicroFlo^™^ Select reagent dispenser (BioTek, Winooski, VT) was then used to add 75 μL MEM + 2 % FBS to all wells of the Stock Sample Plate (Fig. 1A).

Separately, a MicroFlo^™^ Select was used to dispense 30 μL MEM + 2 % FBS to all wells of a 384-well, white, flat-bottom plate (Sample Dilution Plate, Fig. 1B). A SOLO multi-channel robotic workstation with Stacklink plate stacker (Hudson Robotics Inc., Springfield, NJ) was used to transfer 30 μL of the diluted serum from the 96-well Sample Stock Plate in quadruplicate into rows A and I of the 384-well (containing five serum specimens per row, 10 serum samples per plate) Sample Dilution Plate (Fig. 1A–B). A Bravo Liquid Handling Platform (Agilent Technologies, Inc., Santa Clara, CA) was used to execute 30 μL 0.5 log_2_ serial dilutions to create the Serial Dilution Plate (Fig. 1C), from which 5 μL of the diluted serum was transferred into multiple assay plates (Fig. 1D).

### 3.2. Generation of assay plate

A MicroFlo^™^ Select reagent dispenser was used to dispense 20 μL of RD cells (2.5 ×10^5^ cells/mL diluted in MEM + 2 % FBS) to each well of the assay plates containing the serially diluted serum (Fig. 1D). Assay plates were incubated for 30 min at room temperature (15–25 ^◦^C) prior to using a MicroFlo^™^ Select to dispense 10 μL of virus (100 CCID_50_ diluted in MEM + 2 % FBS) to columns 1 through 20. For assay control wells, 10 μL MEM + 2 % FBS was dispensed to columns 21 through 24 (Fig. 1D). Assay plates were covered with a plate lid, wrapped in plastic wrap with a wet paper towel placed at the bottom of the stack, and incubated at 33 ^◦^C, 5 % CO_2_ for 6 days. Final assay volume was 35 μL. A single 384-well assay plate could contain up to ten unique test sera run in quadruplicate, if desired. Up to 160 samples can be tested in a typical 48-plate run.

After a 6-day incubation, ATPlite^™^ luminescence (Perkin Elmer, Shelton, CT) reagent was used to measure intracellular adenosine triphosphate (ATP) as a proxy for viable cell number (Miret et al., 2006). A MicroFlo^™^ Select reagent dispenser was used to add 15 μL of ATPlite^™^ cell lysis buffer and then 15 μL of reconstituted substrate solution to each well. After a 10 min incubation at room temperature (15–25 ^◦^C) in the dark, luminescence was measured on a Victor X4 plate reader (Perkin Elmer, Shelton, CT) configured with two 40-plate stackers and using 0.1 s integration for each well. Raw luminescence counts were exported to custom spreadsheets for analysis. The cutoff for positive/negative wells for neutralization was determined by calculating 80 % of the average luminescence signal for the cell control wells on each plate. A custom macro used luminescent activity to calculate the endpoint titer for each serum dilution where titers of > 3 log_2_ (1:8) were considered to be positive for neutralizing antibodies (Weldon et al., 2016).

## 4. Results

We developed an automated high-throughput EV-D68 microneutralization assay, adapted from the gold-standard 96-well assay (Harrison et al., 2019). The established, traditional 96-well EV-D68 neutralization assay protocol relies on formation of a complete monolayer by RD cells, in order to use visualization and scoring of cytopathic effect (CPE) as the assay readout. Retaining the use of RD cells in the automated assay would be an advantage, since RD cells are highly permissive to EV-D68 infection (Zhang et al., 2019). We shifted to the use of ATPlite^™^ reagent for measurement of cellular viability and assay readout. Traditional reagents like crystal violet often stain cell culture debris in addition to live cells that remain adherent, making it difficult to differentiate dead from live cells (unpublished observation).

**Fig. 2.**
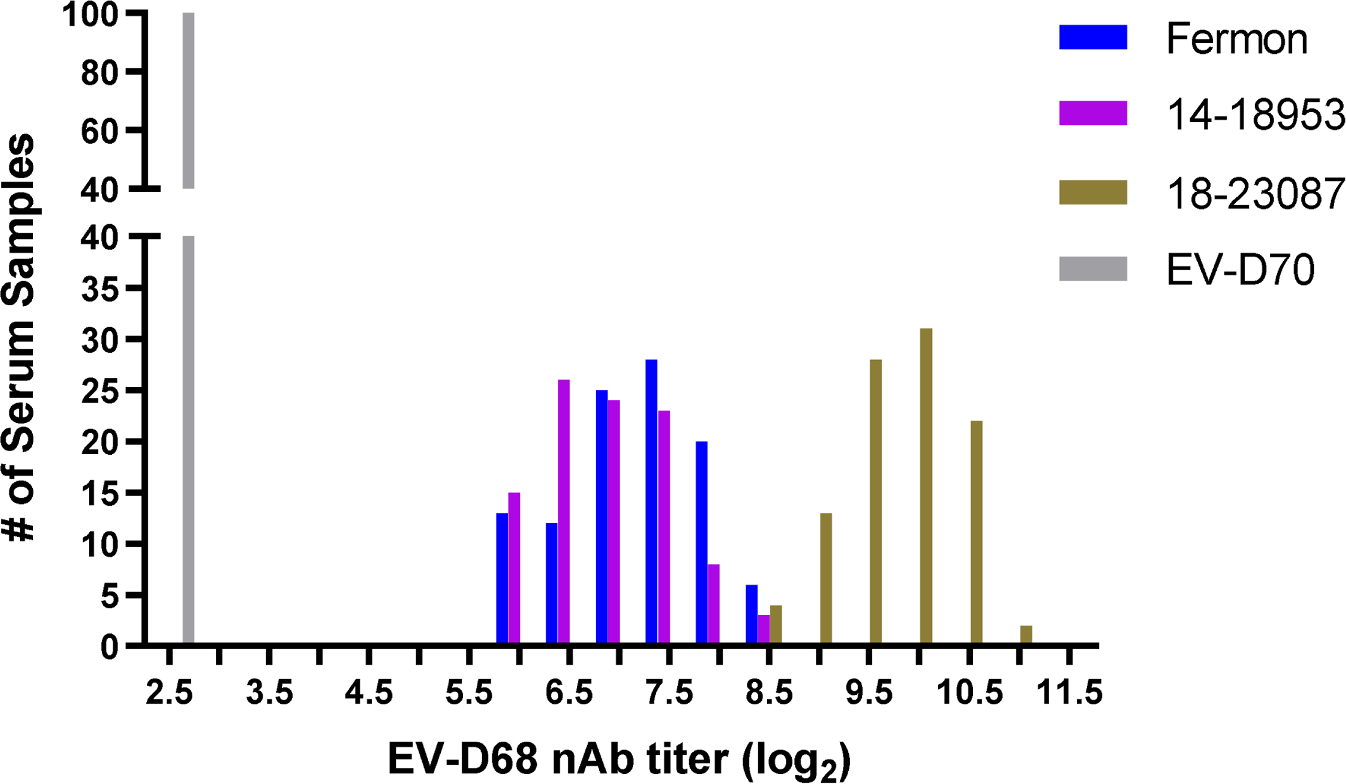
Assay sensitivity and reportable range. One hundred aliquots of human IHRS were used to set and confirm the reportable range of EV-D68 nAb titers for the 384-well assay. IHRS exhibited a wide distribution of nAb titers to EV-D68 strains Fermon, 14–18953, and 18–23087, with 98 % of titers (98/100) falling within a range of 2.5 log_2_ (10^2.5^; the LLOQ) to 10.5 log_2_ (10^10.5^; the ULOQ). EV-D70 served as a negative control with 100 % of the titers (100/100) falling at the LLOQ.

The reportable nAb titer range for the 384-well assay was initially set based on optimization of virus input (100 CCID_50_ titer) for each EV-D68 strain, as well as past experience with the 96-well assay. We used control serum designated as in-house reference serum (IHRS), which is pooled human sera with high neutralizing antibody titers to each EV-D68 strain, to serve as initial test sera to confirm the reportable range (Fig. 2). A dilution scheme of 2-fold serial dilutions, starting with a 1:8 dilution, was used for IHRS replicates, to yield detectable Ab titers falling neatly within a range of 2.5 log_2_ (10^2.5^; lower limit of quantitation [LLOQ]) to 10.5 log_2_ (10^10.5^; upper limit of quantitation [ULOQ]). IHRS exhibited a wide distribution of nAb titers to EV-D68 strains Fermon, 14–18953, and 18–23087. Ninety-eight percent (98/100) of the IHRS replicates tested fell within the set reportable range of antibody titers, from 2.5 to 10.5 log_2_ (Fig. 2) indicating a high probability that tested samples with antibodies against EV-D68 will fall within this range. Consistent with parameters for the poliovirus neutralization assays (Weldon et al., 2016), the titer threshold was set at ≥ 3 log_2_ to consider a sample as positive for nAb activity (as determined by a value ≥ 0.5 log_2_ from negative control antisera). It should be noted, however, that the minimum protective serum antibody titer for EV-D68 is unknown.

**Fig. 3.**
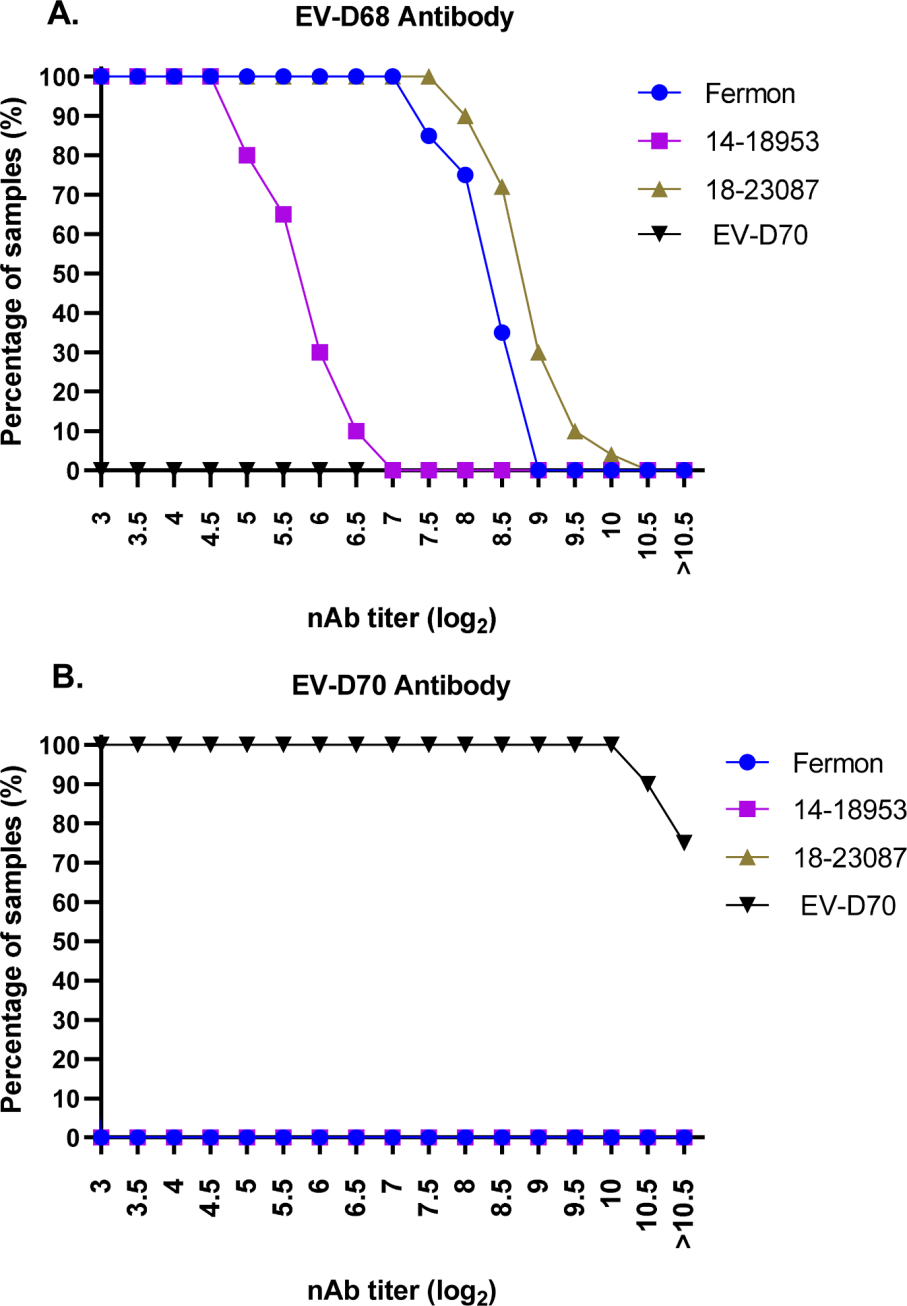
Assay specificity demonstrated by total positive and total negative control samples. A. EV-D68-specific antiserum T2–3420 was assessed as a true positive control (TPC) for use in the 384-well microneutralization assay. Reverse cumulative distribution curves of neutralizing antibody titers are shown against three EV-D68 strains and an irrelevant control virus (EV-D70). The EV-D68-specific antiserum specifically neutralized all three EV-D68 strains while having no neutralizing activity against EV-D70. B. EV-D70-specific antiserum was evaluated as a true negative control (TNC) for use in the assay. Reverse cumulative distribution curves of neutralizing antibody titers are shown against EV-D70 and three strains of EV-D68. The negative control antiserum effectively neutralized EV-D70 and failed to neutralize any of the EV-D68 strains tested.

We next characterized the specificity of potential positive and negative control sera, EV-D68-specific antiserum (T2–3420) and EV-D70-specific antiserum (J670/71), respectively. The two antisera were tested against three EV-D68 strains as well as EV-D70 (Fig. 3). EV-D68 antiserum T2–3420 specifically neutralized all three EV-D68 strains, albeit with differences in the nAb titers against each strain, while no neutralizing activity was detected against EV-D70 (Fig. 3A). Conversely, the J670/71 antiserum raised against EV-D70 neutralized EV-D70 and did not have any neutralizing effect on the three EV-D68 strains tested (Fig. 3B). These data support EV-D68 antiserum T2–3420 as a positive control and EV-D70 antiserum J670/7 as a negative control for the assay.

**Fig. 4.**
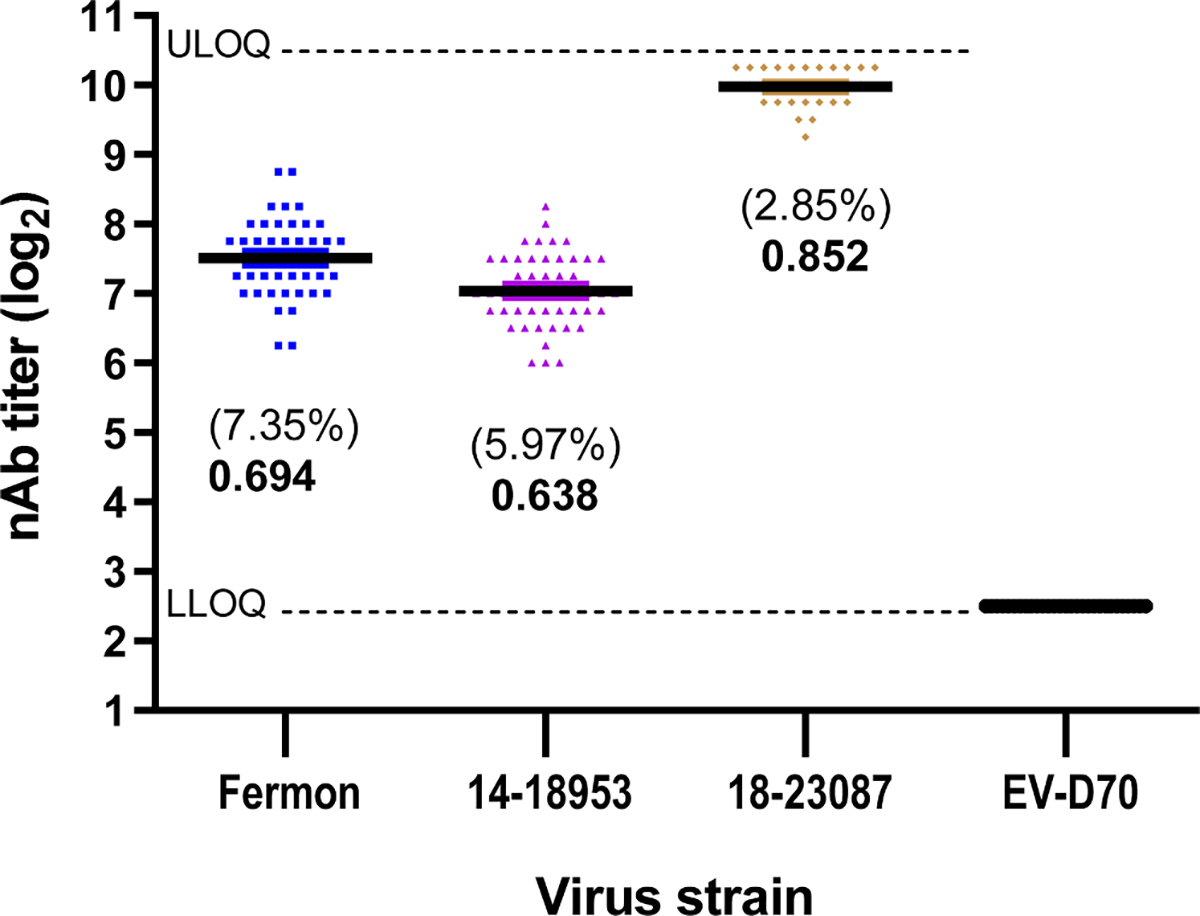
Assessment of assay accuracy and precision. EV-D68-specific nAb titers in 50 replicates of either IHRS or negative control EV-D70 antiserum were determined in the 384-well microneutralization assay using three strains of EV-D68. Individual nAb titers were plotted for each virus strain; the mean for each is indicated by a solid line. Dashed lines indicate the LLOQ and ULOQ. As a measure of accuracy, the percent coefficient of variation ( %CV) was determined. The Z-factor (in **boldface type**) was determined for each viral target as a measure of assay precision.

To assess intra-run variability of the assay, the nAb titers for 50 aliquots of the same IHRS pool were determined against three EV-D68 strains within a single experiment (Fig. 4). The nAb titers were quantified by limiting dilution and endpoint titers calculated. The coefficients of variations, calculated as standard deviation of titers / mean titer x 100, were 7.35 % for EV-D8 strain Fermon, 5.97 % for strain 14–18953, and 2.85 % for strain 18–23087. All three values are well below the recommended cutoff of 20 % CV for accuracy (https://www.fda.gov/files/drugs/published/Bioanalytical-Method-Validation-Guidance-for-Ind ustry.pdf). We used Z-factor analysis (Zhang et al., 1999) as a measure of precision, reliability, and robustness of the assay. For high-throughput screening assays, the recommended Z-factor should be ≥ 0.5 – 1.0. This range indicates that the values of the positive and negative controls are distant enough from each other for high assay reliability. The Z-factors for the nAb titers determined during testing of the 50 samples of IHRS against EV-D68 strains Fermon, 14–18953, and 18–23087 were 0.694, 0.638, and 0.852, respectively (Fig. 4), indicating a high level of precision, reliability, and robustness for the assay.

**Fig. 5.**
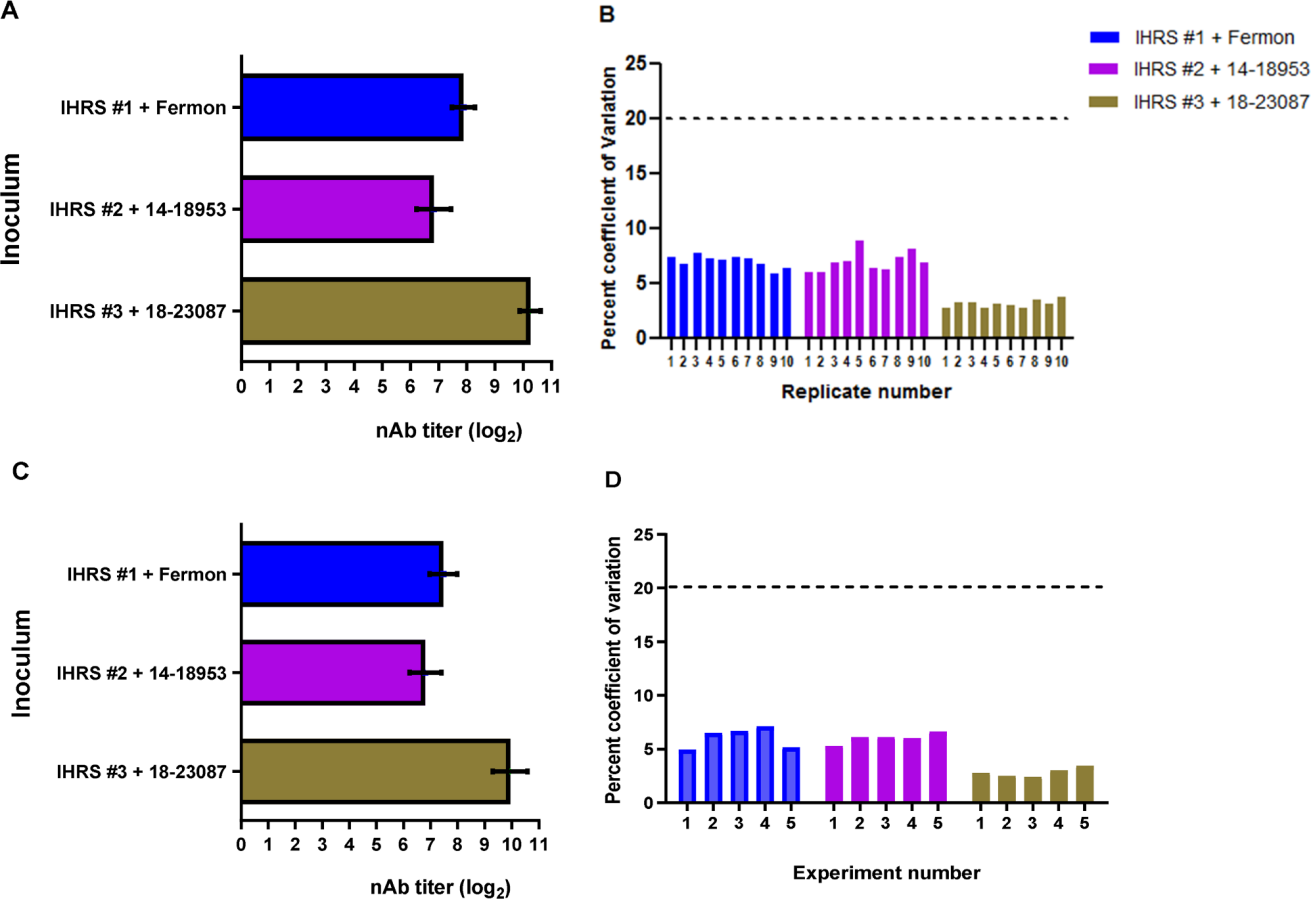
Assessment of intra- and inter-assay reproducibility. A and B. Intra-assay reproducibility. Intra-assay reproducibility was assessed by measuring EV-D68-specific nAb titers (mean and SD) in three separate IHRS aliquots running 10 tests of each in a single run of the microneutralization assay. A different EV-D68 strain was used as the viral target for each of the three IHRS aliquots. Percent CVs were determined from titer results and are shown for each of the 10 intra-assay tests. C and D. Inter-assay reproducibility. Inter-assay reproducibility was determined by 50 tests of 3 separate IHRS aliquots in five independent runs of the microneutralization assay. A different EV-D68 strain was used as the viral target for each of the three IHRS aliquots. Dashed lines indicate the FDA-recommended 20 % CV ceiling for suitable assay reproducibility, with intra- and inter-assay reproducibility falling well below the cutoff.

Replicates of IHRS were tested against each of the three EV-D68 strains. For intra-assay reproducibility,10 replicates were tested within a single run of the assay (Fig. 5A, B) and 50 total replicates in five independent runs were tested to determine inter-assay reproducibility (Fig. 5C, D). Both intra-assay and inter-assay variation are well below the 20 % CV threshold, indicating a high level of reproducibility. Interestingly, EV-D68 strain 18–23087, when tested against IHRS, consistently resulted in higher titers (which were near or at the ULOQ). This may suggest differences in antibody affinity. It should be noted that accuracy, precision and reproducibility, for EV-D68 strain 18–23087, could be positively skewed but remained within an acceptable range.

We explored if EV-D68 antibody T2–3420 could cross neutralize additional enteroviruses. Dilutions of antibody were tested against select viruses (100 CCID_50_ titer). Viruses representing Enterovirus species A (EV-A71), species B (CVB5), species C (PV1) and species D (EV-D68, EV-D70) were assessed using the 384-well microneutralization assay. Of the viruses tested, only species D (EV-D68) viruses exhibited neutralization (Table 1).

**Fig. 6.**
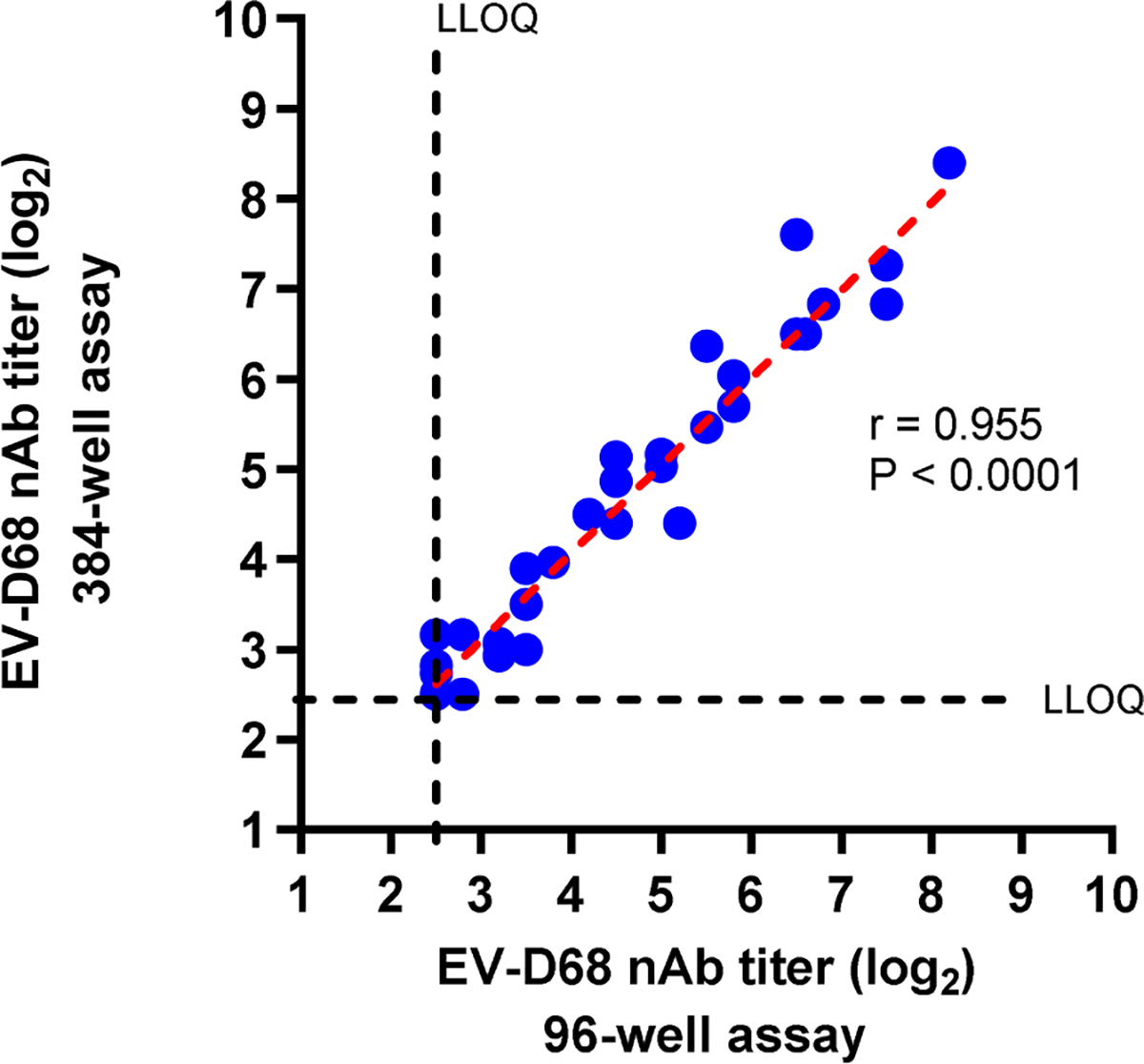
Direct comparison of 96-well and 384-well microneutralization assay formats. Human serum samples (50 total) with known nAb titers against EV-D68 (Fermon strain) were used to compare results from the 96-well versus 384-well assay formats. The same serum samples were tested in parallel in the two assay formats and the nAb titers determined. Dashed lines indicate the lower limit of quantitation (LLOQ) for the assays. P-value was determined by two-way ANOVA using GraphPad Prism.

We directly compared the performance of the 384-well assay to the previously validated and published 96-well assay. Fifty human serum samples were tested in parallel using the two versions of the assay. The resulting nAb titers for the two assays were highly concordant, except a couple of data points at the LLOQ, with a correlation coefficient of 0.955 (Fig. 6).

## 5. Discussion

The 384-well EV-D68 neutralization assay developed here met or exceeded FDA-established guidelines (https://www.fda.gov/files/drugs/published/Bioanalytical-Method-Validation-Guidance-for-Industry.pdf) for sensitivity, specificity, accuracy, precision, and reproducibility. Detection of intracellular adenosine triphosphate (ATP) was used as a proxy of cell viability, allowing for a quantitative measure of virus-induced CPE, which is directly related to the level of protection provided by nAbs present in tested serum samples. This approach is ideal for high-throughput assays due to its homogenous mix-and-measure assay format, use with cell lines independent of their adherence properties, and amenability to high-density plate formats that can be automated.

The choice of instrumentation for a high-throughput automated neutralization assay is important for the performance of the assay. Small-footprint liquid handlers and reagent dispensers allow the use of existing biosafety cabinets, an important consideration for work using cells and infectious agents. User-friendly software and custom-written scripts facilitate a minimal learning curve for end users. Automated liquid handling instruments allow miniaturization of the assay from 75 μL in 96-well plates, to 35 μL 384-well plates, resulting in reagent savings and conservation of specimens. Automation of small volume transfers and 384-well serial dilutions resulted in greater accuracy and precision, reducing opportunities for user error associated with manual transfers (data not shown).

The new assay will facilitate larger studies than what is feasible with the current 96-well assay, including population-based serosurveys that may span multiple years and demographics as well as screening against multiple virus strain targets. The automated platform allows a sample throughput of up to 160 samples against three virus strains per day and has streamlined several labor-intensive steps including serial dilutions, bulk reagent additions, and microplate reading. This flexible, automated assay platform promises to support future high throughput testing of antibodies and small molecule targets against picornaviruses and other cytolytic viruses.

## Figures and Tables

**Table 1:** Cross neutralization of EV-D68 antibody T2–3420.

Virus	Species	Titer log_2_

EV-A71 (SH-17)	A	< 2
CVB5 (Faulkner)	B	< 2
PV1 (Sabin 1)	C	< 2
EV-D68 (Fermon)	D	8.5
EV-D68 (14–18953)	D	5.5
EV-D68 (18–23087)	D	9.5
EV-D70 (J670/71)	D	< 2
